# Interactions of Consanguinity and Number of Siblings with Childhood Acute Lymphoblastic Leukemia

**DOI:** 10.1155/2020/7919310

**Published:** 2020-12-08

**Authors:** Ameer Kakaje, Mohammad Marwan Alhalabi, Ayham Ghareeb, Bahjat Karam, Bassam Mansour, Bayan Zahra, Othman Hamdan

**Affiliations:** ^1^Faculty of Medicine, Damascus University, Damascus, Syria; ^2^Haematology Department, Children's University Hospital, Damascus University, Damascus, Syria

## Abstract

Acute lymphoblastic leukemia (ALL) is a common malignancy in children. Consanguinity has a high prevalence in developing countries and increases the probability of homozygosity for many genes which may affect ALL and its prognosis. We conducted a study to explore the impact of consanguinity and number of siblings on ALL as there are currently no studies to describe this effect. Data were collected from patients' records from the Children's University Hospital of Damascus University, which is the major cancer centre for children in Syria. This study included 193 children with ALL over one year. Number of siblings was not with the French–American–British (FAB) classification, gender, ALL subtype, or risk of ALL children. When comparing consanguinity degrees and complete blood counts at diagnosis, significant contradicting data were found in the third-degree and fourth-degree consanguinity when compared to one another and to not having consanguineous parents as third degree consanguinity was associated with normal platelets but lower WBC counts, and fourth-degree consanguinity was associated with normal haemoglobin levels and WBC counts, but lower platelet counts. Having consanguineous parents was also associated with acquiring ALL at an older age, L2 FAB classification, having a positive family history for malignancies, and not having hepatosplenomegaly (*P* < 0.05). Although L2 is known to be a poor prognosis indicatory, no association was found with consanguinity and risk. Finally, no association was found with ALL subtype or risk (*P* > 0.05). Although consanguinity and number of siblings have affected some variables and prognostic features of childhood ALL, the aetiology is not clear and we need further studies to clarify such an association as this will help in optimising therapy and accurately determine the risk.

## 1. Introduction

Consanguineous marriages are defined as two biologically related people joining in marital union. It is a social tradition that is carried on through generations among certain populations and depends on many factors, including religion, culture, and socioeconomic status [1–4]. The most common form is first cousin marriages with a preference for the offspring of the father's brother, and the married couple in this case shares 12.5% of the genes. Less commonly are double first cousins marriages who share 25% of the genes and first cousins once removed with 6.25% of the genes being shared [5, 6].

Consanguineous marriages are practiced worldwide with a variation in prevalence; in Europe and North America, it is less than 1%. Meanwhile, this rate can reach up to 50% of the general population in Arab countries [7]. For instance, the rate can reach up to 56% in Saudi Arabia [8, 9] and 68% in Egypt [10].

Consanguineous marriages are associated with congenital malformations and recessive gene disorders along with other comorbidities and mortalities [11]. One study in the Middle East found a high prenatal and infant mortality rate with consanguinity [10]. Another study found that 40% of children with cancers meet the criteria for hereditary cancer susceptibility syndromes [12]. Consanguinity also increases the probability of having homozygosity as it increases the rate of acquiring two copies of the defected recessive alleles from the common ancestor [13]. However, there are other studies that described a reverse association between consanguinity and some diseases and cancers [14–16]. This means that consanguinity has complicated interactions that might either increase or decrease susceptibility of certain cancers.

Acute lymphoblastic leukemia (ALL) is the most common diagnosed cancer in children under the age of 15 as it consists around 25% of the cancers [17]. The astonishing progress of treating childhood cancer came as a result of multimodal and adapted treatment strategies depending on the risk. Around 8 to 9 out of 10 of all children who are properly managed are considered cured [18–20].

Many recessive genes that predispose to ALL and other haematological malignancies can be found more frequently in patients with consanguineous parents such as mismatch repair deficiency syndrome [21]. Meanwhile, although having more siblings was found to predispose to many solid tumours such as anal and stomach cancers, other cancers' risk decreased such as melanoma and endometrial cancer [22]. Acute monocytic leukemia and Hodgkin's lymphoma risks increase in large families, particularly in older siblings [23]. However, there are contradicting data about number of siblings' effect on ALL susceptibly [23–26].

ALL in Syria has distinguished features that may be from the unique environment and exposure to different substances [26, 27]. This is the first study to evaluate the effect of consanguinity and number of siblings on different variables of childhood ALL as this is important for more personal treatment to maximise the outcomes, mainly in countries with high prevalence of these phenomena.

## 2. Materials and Methods

### 2.1. Study Design

This is a cross-sectional study conducted in the Children's University Hospital of Damascus University which is the major hospital for haematological disorders in Syria [27]. The data covered the period between 21^st^ August 2017 and 21^st^ August 2018. This hospital is the major paediatric cancer centre among two centres in Syria and provides free healthcare to its patients [27].

### 2.2. Sampling and Data Collecting

This study included children who had ALL and were 14 years old or younger. ALL was diagnosed by bone marrow aspiration and immune phenotyping. Information was obtained from the hospital's records by the medical examining team at the time of diagnoses, and the information was provided by the child's caregiver(s).

Multiple characteristics of patients such as age, gender, and governorate of origin were also recorded in addition to different characteristics of ALL (Tables 1 and 2). Caregivers were asked by hospital physicians about the biological relationship between both the father and the mother and history of cancers and leukemia in the family.

### 2.3. French–American–British (FAB) Classification

FAB classification is based on morphology and cytochemical staining; it remains effective despite the availability of cytogenetics and can add diagnostic accuracy in some cases [28]. A skilled professor in haematology was involved in determining the French–American–British (FAB) classification [29] for each ALL patient whether it was L1 or L2.

### 2.4. Prognostic Risk

We used Berlin-Frankfurt-Münster (BFM) to determine the risk of ALL patients [30]. However, we merged the two groups of standard and intermediate risks as it will be easier to evaluate and they can overlap, mainly due to lack of genetic testings [27].

The risk was assessed based on age, white blood cell (WBC) count at time of diagnosis, cytogenetic changes such as having Philadelphia chromosome, having medical comorbidities, cerebrospinal fluid (CSF) analysis, testicular involvement, inability to tolerate standard chemotherapy, response to initial therapy, predicted outcome, organomegaly, and lymphadenopathy on examination or chest X-ray (CXR); these were all considered in the determination of each patient's prognosis to conduct a correct chemotherapy protocol [31].

### 2.5. Other Variables and Definitions

Consanguinity was defined as third-degree relatives (first cousins) and fourth-degree relatives (second cousins, second cousins once removed). Family history was obtained based on the family of the subjects having malignancies in their families regardless of type of cancer or age of presentation. All findings are at time of diagnosis.

Constitutional symptoms were defined as having fever, anorexia, weight loss, or fatigue. CXR was considered positive when it had a mediastinal enlargement or hilar lymphadenopathy. WBC count of 1500-11 500 × 10^9^ cells/L, haemoglobin level of 11-16 g/dL, and platelet counts of 150 000-400 000 × 10^9^ cells/L) were considered normal.

Educational levels were divided into 3 subgroups according to the highest obtained degree. Low educational level included elementary education or lower. Medium educational level included finishing year 9 or year 12. Finally, high educational level included having a university degree or higher. This system is used in Syria as these groups have distinguished features, and it was used previously in many studies [26, 27, 32, 33].

Genetic testings could not be obtained due to unavailability to these tests in many cases in Syria [27]. The issue for proper funding has affected many studies and rendered it difficult to conduct proper diagnostic tools and to carry on studies other than observational [26, 34]. For easier and more reliable comparisons, only pre-B-ALL and pre-T-ALL were included. We also excluded patients with L2 (Burkitt) leukemia as they may have a confounding viral and/or genetic aetiology that will make the results harder to understand.

### 2.6. Consent and Ethical Approval

Informed consent was taken before using and publishing their data. The study was approved by the ethic committee of Damascus University.

### 2.7. Data Analysis

Data were processed using IBM SPSS software version 25 for Windows (SPSS Inc., IL, USA). Chi-square, one-way analysis of variance (ANOVA), and independent *t*-test were performed to determine the statistical significance between the groups. We calculated odds ratio (OR) and the 95% confidence intervals for the groups using the Mantel–Haenszel test by using the same software. Values of less than 0.05 for the two-tailed *P* values were considered statistically significant.

## 3. Results

**Figure 1:**
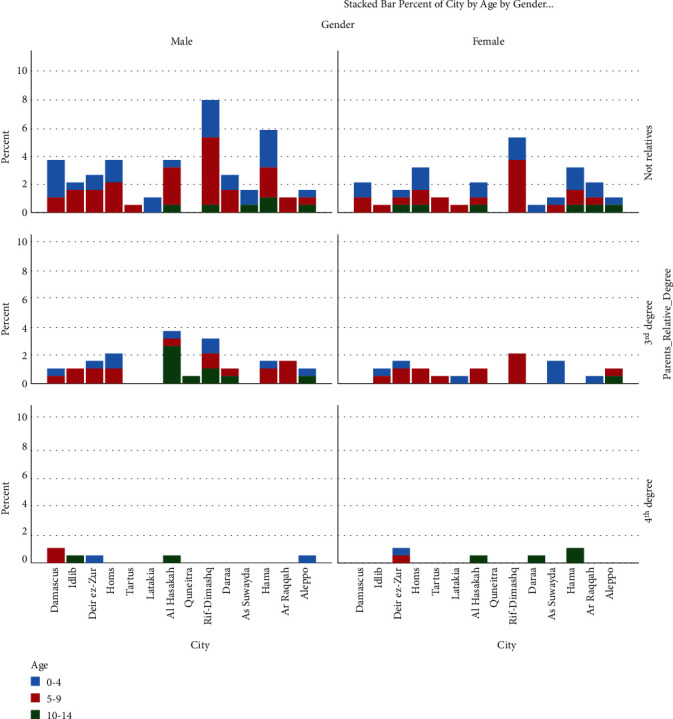
Showing percentages of children with ALL in each city with their consanguinity degree, age, and gender.

Our sample had 193 ALL patients and their characteristics are demonstrated in Tables 1 and 2. The distribution of children with ALL in each city with their consanguinity degree, age, and gender is shown in Figure 1. Our sample had a mean number of siblings of 4.8 ± 2.7.

### 3.1. Number of Siblings

When comparing number of siblings in ALL children with other variables (Table 3), we found that having a mother of a lower educational level is correlated with having more siblings in ALL patients (*P* = 0.0002). Having more siblings is also associated with ALL diagnosis at an older age (*P* = 0.0245) when comparing age groups 10-14 with 5-9 years, and *P* = 0.0031 when comparing age groups 5-9 with 0-4 years. However, when comparing gender, place of living, consanguinity, main presenting symptom, lymphadenopathies, hepatosplenomegaly, haemoglobin level, WBC count, CXR findings, ALL subtype, CD10 levels, prognostic risk, and FAB classification with number of siblings, there was no statistically significant difference (*P* > 0.05). Nevertheless, we found *P* = 0.079 when comparing number of siblings with consanguinity overall. The mean number of siblings for patients with consanguineous parents was 5.26 ± 2.483 (*P* > 0.05) when comparing different consanguinity degrees with number of siblings.

### 3.2. Consanguinity and Complete Blood Count (CBC)

Consanguinity rate was 36.3% with 95% confidence interval of 29.5%-42.5%. Comparing consanguinity with ALL characteristics is demonstrated in (Table 4). Although having parents with third-degree consanguinity was associated with a higher WBC count compared to nonconsanguineous parents (*P* = 0.0457; OR, 1.967; 95% CI, 1.008-3.839), having parents with fourth-degree consanguinity was correlated with having a normal WBC count (*P* = 0.0405; OR, 4.566; 95% CI, 0.649-22.222). When comparing patients with parents of third-degree consanguinity and fourth-degree consanguinity, we found a significant difference in WBC count as it was higher in the third-degree consanguinity group (*P* = 0.0029; OR, 0.111; 95% CI, 0.022-0.569), suggesting a different effect from third and fourth degree on WBC count.

Likewise, when comparing third degree-consanguinity and fourth-degree consanguinity with having a normal or abnormal WBC count, we found similar findings as third-degree consanguinity had an abnormal WBC count more frequently (*P* = 0.0018; OR, 9.709; 95% CI, 1.908-50.000). Patients with third-degree consanguinity had higher rates of abnormal WBC compared to those with no consanguineous parents (*P* = 0.0303; OR, 2.063; 95% CI, 1.065-3.996). In contrast, when patients had parents with fourth-degree consanguinity, they had normal WBC more frequently than those who had parents with no consanguinity (*P* = 0.0357; OR, 4.717; 95% CI, 0.980-22.727).

In ALL patients with low platelet counts (less than 150 000 × 10^9^ cells/L), when comparing third-degree and fourth-degree consanguinity, there was a statistically significant difference as the latter was associated with having platelet counts less than (20 000 × 10^9^ cells/L) (*P* = 0.0433). Patients with third-degree consanguinity had higher platelet counts (more than 400 000 × 10^9^ cells/L) than those with no consanguineous parents (*P* = 0.0404; OR, 10.500; 95% CI, 0.844-130.663). Finally, patients with third-degree consanguinity had more frequently normal haemoglobin level than abnormal haemoglobin level (*P* = 0.0485; OR, 2.433; 95% CI, 0.986-6.024).

### 3.3. Consanguinity and Age

Having consanguineous parents is correlated with a higher incidence of ALL in the oldest age group (10-14) when compared with the 5-9 age group (*P* = 0.0242; OR, 2.619; 95% CI, 1.117-6.138), especially when comparing fourth-degree consanguineous parents and nonconsanguineous parents (*P* = 0.0006; OR, 10.167; 95% CI, 2.228-46.384). When comparing age groups, the oldest group (10-14 years) had more parents with fourth-degree consanguinity than the younger group (5-9 years) who had parents with third-degree consanguinity more frequently (*P* = 0.0223; OR, 5.455; 95% CI, 1.159-25.662). In patients aging 10-14 years, male gender was associated with third-degree consanguinity (*P* = 0.0332; OR, 10.00; 95% CI, 0.957-100).

### 3.4. Consanguinity and FAB Classification

Having consanguineous parents is also correlated with having more patients with L1 FAB classification than patients with nonconsanguineous parents (*P* = 0.0054; OR, 2.700; 95% CI, 1.329-5.487). This was the same when comparing nonconsanguinity with third-degree consanguinity (*P* = 0.0079; OR, 2.750; 95% CI, 1.290-5.865). Moreover, having consanguineous parents was associated with not having hepatosplenomegaly (*P* = 0.0161; OR, 2.242; 95% CI, 1.153-4.367), similarly to fourth-degree consanguinity (*P* = 0.0271; OR, 3.676; 95% CI, 1.092-12.346).

L2 FAB classification was also found more frequently in male patients with fourth-degree consanguinity (*P* = 0.0054). In patients with L2, ALL incidence was higher among male patients with consanguineous parents (*P* = 0.0378). However, in patients with L1, ALL incidence was higher among female patients with fourth-degree consanguinity (*P* = 0.0432).

### 3.5. Family History

**Figure 2:**
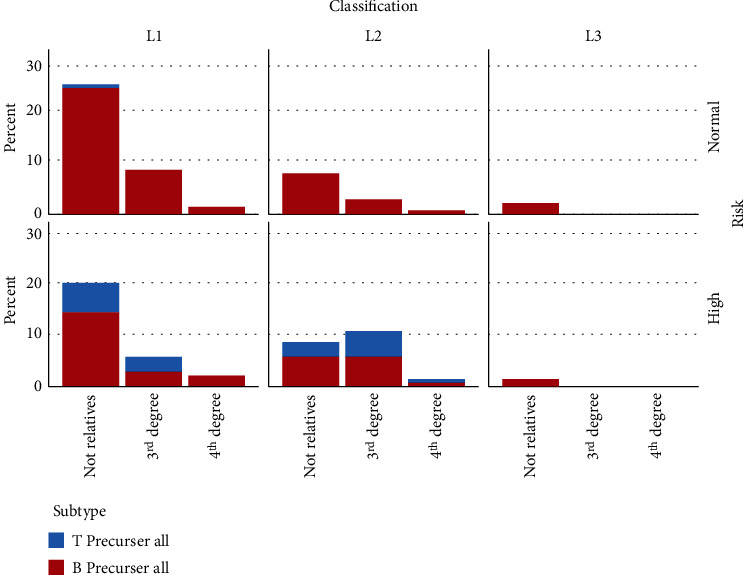
Showing the risk of ALL patients, their subtype, and consanguinity degree.

Having a positive family history was more frequent in children with parents of fourth-degree consanguinity (*P* = 0.0387; OR, 4.333; 95% CI, 0.975-19.255). The risk of ALL patients, their subtype, and consanguinity degree are demonstrated in Figure 2. In male patients, having consanguineous parents was correlated with having positive family history (*P* = 0.0446) and with having abnormally high platelet counts when diagnosed (higher than 400 000 × 10^9^ cells/L, *P* = 0.0088).

### 3.6. Other Variables

Having consanguineous parents in ALL children was not found to be correlated with ALL subtype, prognostic risk, duration of symptoms before evaluation, CXR findings, parents' educational level, and CD10 (*P* > 0.05). When adjusting for age, gender, subtype, or FAB classification, no other statistically significant differences were found when comparing patients with consanguineous and nonconsanguineous parents.

## 4. Discussion

Consanguinity is noted in many successive generations and children born to a consanguineous family are more likely to marry one of their relatives and carry on this tradition. This pattern is reported in many neighbouring countries to Syria in the Middle East and other parts of Asia such as Turkey [35], Iran [36] and many North African countries. This leads to higher rates of homozygosity for many genes.

In this study, in the 193 ALL patients, 30.1% had third-degree consanguineous parents, 6.2% had fourth-degree consanguineous, and consanguinity overall was 36.3% which is higher than what was reported in Tehran in ALL children (15.7%) [37] (*P* = 0.0001), but lower than Saudi Arabia (41.7%) [12] (*P* > 0.05) and the UAE (80%) [38] (*P* < 0.0001). We also found that having a mother with a lower educational level is associated with consanguinity which is found in multiple studies [6, 39–41]. However, we did not find this association with the father's educational level in our study.

This study covered many variables of childhood ALL that accompanied consanguinity and number of siblings. As this is the first research to study their effect on different variables, we do not have a rational explanation for many of the findings.

### 4.1. Childhood ALL and Number of Siblings

The mean number of siblings measured is 4.81 ± 2.7 which seemed an unusually high number of siblings at time of diagnosis. Lower educational levels in mothers were noted with higher number of siblings. Furthermore, the higher number of siblings was more common among the older population (aged 10-14) when compared to the younger one (0-9) years. However, as having many siblings requires the mother to have a shorter age interval between births of siblings, most of ALL patients in our study had this unusually short interval which may indicate that having many siblings could be indirectly associated with ALL. This contradicts one study which found a significant increase in risk for childhood ALL with long age intervals between births [24]. Another study found that young siblings had lower risk compared to the older ones [23]. However, a study from Denmark did not find a correlation between ALL and age intervals [25]. One case-control study in Syria found no association between number of siblings and ALL [26].

One theory which may explain the increased risk of some cancers when having more siblings is that early-onset and inherited diseases may be demonstrated more frequently in small families due to selection and the limitation of the reproductive period of the parents [22]. In contrast, it was speculated that the decreased risk in some cancers such as testicular cancer can be explained by the exposure hypothesis. This hypothesis indicates that in utero exposure to oestrogen will be higher in early pregnancies compared to later ones [22].

We found a slight insignificant increase in number of siblings in patients with consanguineous parents, and there was no difference between different consanguineous groups. This is similar to a study done in Qatar which found a higher fertility in all consanguineous groups and the mean number of pregnancies was also slightly higher in first cousin unions [39]. Risk of ALL was also associated in some studies with high educational level of the mother [25] as they postpone having their first child. However, it was inconclusive if birth order affected ALL. Increased number of siblings in ALL children was correlated with acquiring ALL in older patients (Table 3) (*P* < 0.05). However, it was not correlated with gender, FAB classification, CD10, WBC and platelet counts, haemoglobin CXR findings, ALL subtype, and risk.

### 4.2. Complete Blood Count and Consanguinity

In Syria, only 2% of childhood ALL were found to have normal WBC and platelet count [27]. Having conflicting results between patients with third- and fourth-degree consanguinity in parents may reflect overlapping factors being involved. This is especially noted for WBC counts as it is a prognostic indicator and was also suggested to be made a determinant factor for the higher risk group [42].

High frequency of abnormal WBC count was found in patients with third-degree consanguinity, but high frequency of normal WBC count was found in patients with fourth-degree consanguinity; this was reversed when comparing platelet counts as lower platelet counts were found with fourth-degree consanguinity, and normal platelet counts were found more frequently with third-degree consanguinity. Normal haemoglobin levels were also found more frequently with fourth-degree consanguinity.

This all suggests that third-degree consanguinity is correlated with factors that lead to having higher platelets counts but lower WBC counts and that fourth-degree consanguinity is associated with factors that lead to having more normal haemoglobin levels and WBC counts, but lower platelet counts.

### 4.3. FAB Classification and Consanguinity

L2 in Syria was found to be higher than most studies [27]. L2 was found more frequently in patients with consanguineous parents, especially in third-degree consanguineous parents mainly in males. FAB classification remains an adequate method especially in developing countries as it does not require expensive tests and can be easily applied in most laboratories [27, 43]. Furthermore, L2 was found to have higher relapse rates and poorer survival [44] which means having consanguineous parents may be associated with poorer prognosis.

### 4.4. Other Factors and Consanguinity

Having fourth-degree consanguinity is correlated with acquiring ALL at an older age 10-14 when compared to third degree or nonconsanguineous parents. Having consanguineous parents of any degree was associated with not having hepatosplenomegaly. Having a positive family history was associated with consanguinity which may indicate a family history of hereditary recessive cancer genes.

Having CD10 marker is a good prognostic indicator, and when CD10 is negative, there is a higher probability of relapse and lower remission rate [45]. However, no statistically significant difference was found when comparing CD10 in patients with consanguineous and nonconsanguineous parents.

Although no direct prognostic risk was associated with either having consanguineous or nonconsanguineous parents, a link was established with having hepatosplenomegaly, having L2, abnormal platelet counts, haemoglobin, and WBC counts at time of diagnosis, higher number of siblings, and lower educational levels in patients of consanguineous parents which all may indicate that consanguinity might worsen the prognosis, either directly or indirectly. However, lower educational level, which is associated with consanguinity and lower education, may be linked to behaviours that may expose children to leukaemogenics [26].

In conclusion, consanguinity and number of siblings have a complex association with ALL. More studies are required to determine the underlying effect and whether there are genes that have not been discovered yet that were involved, or there are other aetiologies or confounding factors that are still unknown.

## Figures and Tables

**Table 1:** Characteristics of children with ALL in Syria.

Characteristic	Count (*n* = 193)	Percentage (%)
Age		
0–4	70	36.3
5–9	94	48.7
10–14	29	15
Missing	0	
Gender		
Male	119	61.7
Female	74	38.3
Missing	9	
Place of living		
Damascus, Rif-Dimashq, and Aleppo	60	32.3
Homs and Hama	41	22
Al-Jazira region	49	26.3
Southern Syria	18	9.6
Syrian coast	8	4.4
Idlib	10	5.4
Missing	7	
Mother education level^∗^		
Low	88	56.4
Medium	52	33.3
High	16	10.3
Missing	37	
Father education level^∗^		
Low	86	53.8
Medium	47	29.4
High	27	16.9
Missing	33	
Consanguinity		
No	123	63.7
Third degree	58	30.1
Fourth degree	12	6.2
Missing	0	

**Table 2:** Characteristics of ALL in children in Syria.

Characteristic	Count (*n* = 193)	Percentage (%)
Main presenting symptom		
Constitutional symptoms	137	75.7
Lymphadenopathy	20	11
Hepatosplenomegaly	5	2.8
Bruising	13	6.7
Accidental	6	3.3
Missing	12	
Hepatosplenomegaly:		
Positive	133	72.7
Negative	50	27.3
Missing	10	
Lymphadenopathy		
Positive	158	81.9%
Negative	33	17.1%
Missing	2	
WBC when diagnosed		
1500 and less	5	2.6
1500–11 500	87	45.5
11 500 and above	99	51.8
Missing	2	
Haemoglobin levels when diagnosed		
11–16	23	12
11–7	101	52.6
7 and less	68	35.4
Missing	1	
Platelets count		
More than 400 000	4	2.1
150 000 to 400 000	21	11.1
150 000 to 50 000	60	31.6
50 000 to 20 000	58	30.5
Less than 20 000	47	24.7
Missing	3	
CXR		
Mediastinal enlargement or lymphadenopathies	32	16.6
Negative	135	80.8
Missing	26	
ALL subtype		
B ALL	151	79.9
T ALL	38	20.1
Missing	4	
CD 10		
81% and more	97	57.1
21% -80%	36	21.2
20% and less	37	21.8
Missing	23	
Prognostic risk		
Standard	91	51.4
High	86	48.6
Missing	16	
FAB classification		
L1	90	62.9%
L2	53	37.1%
Missing	50	

WBC count of 1500-11 500 × 10^9^ cells/L, haemoglobin level of 11-16 g/dL, and platelet counts of 150 000-400 000 × 10^9^ cells/L were considered normal.

**Table 3:** Comparing number of siblings in ALL children with other variables.

Characteristic	Mean number of siblings ± SD	P value
Father education		
Low	5.22 ± 2.77	0.100
Medium	4.21 ± 2.44	
High	4.52 ± 2.59	
Mother education		
Low	5.50 ± 2.77	0.0002
Medium	3.94 ± 2.16	
High	3.31 ± 2.02	
Platelet		
Normal	3.80 ± 1.85	NS
Abnormal	4.93 ± 2.80	
WBC		
Normal	4.84 ± 2.82	NS
Abnormal	4.79 ± 2.65	
Haemoglobin		
Normal	4.26 ± 1.74	NS
Abnormal	4.89 ± 2.83	
Consanguinity		
Negative	4.54 ± 2.81	NS
All	5.26 ± 2.48	NS
Third degree	5.25 ± 2.56	NS
Fourth degree	5.33 ± 2.19	
Age groups		
0–4	3.82 ± 2.32	0.0031
5–9	5.06 ± 2.72	
5–9	5.06 ± 2.72	
10-14	6.39 ± 2.70	0.0245

**Table 4:** Comparison of characteristics of ALL children compared with consanguinity.

	Nonconsanguineous	All-degree consanguinity	*P* value^a^	3^rd^ degree consanguinity	*P* value^a^	4^th^ degree consanguinity	*P* value^a^
Gender							
Male	76	43	NS	37	NS	6	NS
Female	47	27		21		6	
Subtype							NS
B-cell ALL	100	51	NS	41	NS	10	
T-cell ALL	21	17		15		2	
Prognosis risk							
Low	64	27	NS	22	NS	5	NS
High	50	36		31		5	
Duration of symptoms before evaluation							
2 weeks or less	34	16	NS	16	NS	0	0.0645
2–4 weeks	38	21		17		4	
4 weeks and more	48	32	NS	24	NS	8	0.0747
WBC							
Low	2	3	NS	3	0.0656	0	NS
Normal	60	27		18		9	
High	61	38	NS	36	0.0457b	2	0.0405^b^
Haemoglobin level							
Normal	11	12	NS	11	0.0515c	1	NS
Low	68	33		27		6	
Very low	44	24	NS	19	NS	5	NS
Platelets count							
400 000+	1	3	NS	3	0.0404	0	NS
150 000–400 000	14	7	NS	4	NS	3	NS^d^
150 000 and less	108	57		48		9	
CXR							
Normal	92	43	NS	35		8	NS
Abnormal	20	12		10		2	
Age							
0–	50	20	NS	17	NS	3	NS
5–9	61	33		30		3	
10-14	12	17	0.0242	11	NS	6	0.0006
Mother education level							
Low	46	42	NS	34	NS	8	0.0622
Medium	35	17		16		1	
High	12	4	NS	3	NS	1	NS
Father education level							
Low	53	33	NS	26		7	NS
Medium	27	20	NS	18	NS	2	NS
High	17	10		9		1	
CD 10							
Negative	20	17	NS	14	NS	3	NS
20% and above	89	44		37		7	
FAB classification							
L1	65	25	0.0054	20	0.0079	5	NS
L2	26	27		22		5	
Hepatosplenomegaly							
Negative	25	25	0.0161	19	0.0550	6	0.0271
Positive	92	41		35		6	
Lymphadenopathy							
Negative	23	10	NS	7	NS	3	NS
Positive	100	58		49		9	
Family history							
Negative	104	56	0.0811	48	NS	8	0.0387
Positive	9	11		8		3	

ALL: acute lymphoblastic leukemia; NS: not significant; FAB: French–American–British classification. Different total subjects between groups occur as some data is missing. ^a^All *P* values are compared with the nonconsanguineous group. ^b^The *P* value when calculated between normal WBC and abnormal is 0.0303 for 3^rd^ degree and 0.0357 for 4^th^ degree consanguinity. ^c^The *P* value when calculated between normal haemoglobin and abnormal is 0.0485. ^d^The *P* value when calculated between 150 000 and 20 000, and 20 000 was 0.0156, WBC count of 1500-11 500 × 10^9^ cells/L, haemoglobin level of 11-16g/dL, and platelet counts of 150 000-400 000 × 10^9^ cells/L) were considered normal.

## Data Availability

Data will be made available upon reasonable request.
